# Innovations that reach the patient: early health technology assessment and improving the chances of coverage and implementation

**DOI:** 10.3332/ecancer.2016.683

**Published:** 2016-10-28

**Authors:** WH van Harten, VP Retèl

**Affiliations:** 1 The Netherlands Cancer Institute, PO Box 90203, 1006 BE Amsterdam, The Netherlands; 2 University of Twente, Department of Healthcare Technology and Services Research, PO Box 217, 7500 AE Enschede, The Netherlands; 3 Rijnstate Hospital, PO Box 9555, 6800 TA Arnhem, The Netherlands

**Keywords:** coverage with evidence development, health technology assessment, tumour infiltrating lymphocytes, high-dose chemotherapy

## Abstract

With growing concerns for the sustainability of the financial burden that health care—and especially cancer services—poses on the national budgets, the role of health economic analyses in coverage decisions is likely to grow. One of the strategies for the biomedical research field—also in oncology research—to foster coverage and health system implementation, is to anticipate this new role and to involve health technology assessment techniques earlier in various stages of translational research.

In this article, we elaborate on the early involvement of health technology assessment in translational research and the concept of Coverage with Evidence Development in The Netherlands Cancer Institute and give two case examples that are currently ongoing: (1) tumour infiltrating lymphocytes therapy for metastatic melanoma; and (2) high-dose chemotherapy for BRCA1-like subgroup in triple-negative breast cancer.

We conclude with recommendations for institutional policy.

## Introduction

When innovations resulting from research reach the patient in routine clinical practice, the full cycle of translational biomedical research is completed. In the case of innovative procedures, inclusion in guidelines by professional agencies and coverage decisions by policy makers are essential to complete the process and present themselves often as last hurdles, before wider implementation in the clinic and diffusion in the health system is possible.

Coverage decisions are usually made by medical boards within Ministries of Health or within health insurance agencies or health insurance governance and/or advisory boards, such as National Institute for Health and Care Excellence (NICE) in the United Kingdom (www.nice.org.uk). Decisions are mostly taken based on a mix of data, primarily on clinical effectiveness and clinical utility, sometimes in combination with health economic criteria such as cost-effectiveness analyses. The regulations for the latter are often stipulated by those health insurance agencies/boards mentioned earlier and clinicians often have little involvement. Particularly, in the field of drug research, pharmaco-economics has developed into a plethora of techniques under the umbrella of health technology assessment, such as budget impact analysis, cost utility analysis, cost-effectiveness analysis and value of information analysis, with which clinicians often have little affinity. Inclusion of innovative technology in professional guidelines is another essential condition that is depending on factors like strength of evidence, the voice of key opinion leaders and barriers and facilitators related to practical implementation in the clinic. Increasingly, professionals are aware of this aspect and cost-effectiveness or value related aspects are included in their decision process towards adaptation of guidelines. The focus of this paper will be on coverage and system acceptance issues.

With growing concerns about the sustainability of the financial burden that health care—and especially cancer services—poses on the national budgets [1], the role of health economic analyses in coverage decisions is likely to grow. One of the strategies for the biomedical research field—also in oncology research—to foster coverage and health system implementation, is to anticipate this new role and to earlier involve health technology assessment techniques in various stages of translational research.

In this article, we elaborate on the early involvement of health technology assessment in translational research and the concept of coverage with evidence development in The Netherlands Cancer Institute and give two case examples that are currently ongoing; (1) tumour infiltrating lymphocytes therapy for metastatic melanoma; and (2) high-dose chemotherapy for BRCA1-like subgroup in triple-negative breast cancer. We conclude with recommendations for institutional policy.

The Netherlands Cancer Institute is the only accredited, freestanding Comprehensive Cancer Center in The Netherlands, with a large research laboratory, hosting a number of core facilities, and a cancer hospital providing all relevant diagnostic, intervention, and radiotherapy treatment facilities.

## Early-stage health technology assessment

In early stages of technology development, there is usually high levels of uncertainty around various aspects of the procedures or the interventions that are being developed. It is often not exactly known what indications will be relevant, how the technique interferes with the existing methods, what the exact domain or market share (in terms of diffusion), will be.

By verifying a broad range of assessment aspects, such as organisational, ethical, legal, and patient-related factors, it is possible to identify the potentially relevant issues and improve the chances of actual clinical acceptance in an early stage of the process. Examples of this method can be found in studies on nanotechnology and gene array testing in breast cancer treatment [2, 3]. This can assist the researcher and product developers in identifying critical aspects that can influence or facilitate ultimate implementation and acceptance by professionals.

There are various techniques to anticipate on uncertainties related to those aspects. Scenario analysis is an example in which experts are involved to give their best estimate of possible future development of a future technology [4, 5] and related to the aim of coverage, these can produce estimates of possible cost-effectiveness or criteria for the technology in terms of sensitivity and specificity and indication fields in order to become cost-effective. Preferably, the patient’s view should also be incorporated into these steps. It is, however, sometimes difficult for patients to see through all practical hurdles and consequences, when confronted with early-stage technology of which the exact indication ranges and effects are not yet known. Scenario analysis is an example in which experts are involved to give their best estimate of possible future development of a future technology [4, 5].

Modelling techniques in which a decision algorithm of treating the decease is adapted by the data on the new technology characteristics can be used to estimate health economic values. This can be either used to stepwise improve the model when additional data become available [6], but also to provide conditional specifics on, for example, sensitivity and specificity or costs ranges that should be applicable for the technology to have success [7]. Value of information (or value of further research) analysis is used to calculate the possible value of investing in further research for society giving an impression on the relation between *a priori* investments versus societal returns [8]. Applying these in early stages can inform researchers and research policy makers on the sensibility to continue a certain research line or adapt it according to its chance of clinical adoption/acceptance. It could be seen that economic analysis in early stages threatens the freedom of the fundamental research process, but as clinical diffusion becomes an explicit target, the results of the health economic modelling should be seen as supportive to the decisions to be made, and it can be wise to take this information into account when deciding on the progress of research lines.

If there is wide consensus among key opinion leaders on the clinical effectiveness and clinical utility of a new technology, cost-effectiveness analyses usually provide straight forward answers. In those cases, it is merely up to the decision boards to finalise the coverage decision process according to established criteria.

## Coverage with evidence development

Increasingly, countries realise that there are often flaws in the data on new technologies, which—when promising in terms of health-added value—pose a dilemma for the regulators in that a promising technology may be withheld from the population when a coverage decision is delayed due to a late start of cost-effectiveness analysis and/or missing information.

Many governments are experimenting with coverage evidence development (CED) or comparable schemes [9]. The arrangement aims to solve the problem of providing fast access of promising new medical technologies, while the underlying data may not be complete in all aspects. Often this means a period of formal introduction and coverage by the national health insurance or by special government funding, in combination with prospective or controlled research and registration (and follow-up) of patient data. Alternatives that are especially considered in the case of expensive new cancer drugs are ‘pay for performance’, ‘no-cure no-pay’, or ‘risk-sharing’ schemes [10].

In The Netherlands, the government has experimented with an early schedule of CED in which initially a few procedures were included combined with rather large funding of research programs on those technologies. Establishing the prognostic value using a 70-gene microarray technique (MammaPrint) for node-negative breast cancer was included as a pilotCED; a prospective feasibility trial including data management, diagnostic testing and evaluation, and an early technology assessment (constructive technology assessment) was funded. It lead to positive decisions of various individual health care insurances. A lack of data from a controlled randomized trial, however, hampered diffusion and a formal coverage decision was delayed until data from controlled studies would be available. In view of trials with personalized treatment on biologically plausible targets, we expect that prospective cohort based trials or mixed designs—as examples of innovative trial funding will gain ground, as the need for innovative trials with small sub groups of patients is expected to grow in the coming years, considering the rapid developments in Personalised Medicine.

## CED scheme in The Netherlands

After this phase of experiments, a formal procedure to decide on CED was installed by law in The Netherlands as from 2012 for hospital based new technologies. The procedure to be followed means that in year 0, a call will be posted with a deadline of 1st September for possible procedures to be included in the CED programme. This will require information on (A) the current state-of-the-art regarding usual care and the new technology, clinical effectiveness, and clinical utility, a first estimation of the cost price of the new technology, and on the position and (preferably) support from the relevant scientific medical societies and data on the health economic status and (B) a full-research proposal including a randomised controlled trial and a health technology assessment, with a clearly formulated research question regarding the (cost)effectiveness of the new intervention. This should be conducted and finalised in a period of around 4 years in order to finalise the files for a final coverage decision. In year 1, a preliminary decision is taken on the innovative treatments/procedures that may qualify for CED. First, the Dutch health care insurance board advises based on feasibility and societal relevance, second, the trial funding agency advises based on the quality of the proposals, and finally, the ministry of health makes a final selection for potential candidates for the CED programme. In the second half of year 1 and the first half of year 2, the design is further elaborated and approved and an agreement with all stakeholders various aspects of service delivery are negotiated (such as an exit strategy in case of inferior results). Finally, after final approval of the ministry of health, the CED trial can start usually in year 2. Throughout the study period, every 6 months, there is a progress meeting including all involved stakeholders, to closely monitor the process. After finalising the trial (3,5 years), a certain period (~6 months) is allowed to further cover the procedures awaiting the finalization of the research and presenting the final dossier to the insurance board and the ministry. At that moment, if the new intervention meets the criteria of ‘the state of science and practice’, a final decision on coverage can be taken. In the application stage, the view of professional medical societies is involved as well as in the ultimate decision on the consequences of the project’s findings; in this way, swift acceptance in updating guidelines with the new technology is assured both in the CED and in the following national implementation phases.

Box 1. Tumour-infiltrating lymphocytes (TIL) therapy for metastatic melanoma [16, 17].

Within melanoma metastases lymphocytes can be found. These tumour-infiltrating lymphocytes (TIL) have become less effective due to immunosuppressive functions from the tumour, or the tumour microenvironment. It is possible to excise a metastasis from a patient and extract TIL from it. The TIL can then be cultured and multiplied to large numbers. After a cycle of non-myeloablative chemotherapy, the TIL are given back to the immunosuppressed patient, followed by infusion of high-dose bolus interleukin-2. In around 50% of patients treated with TIL, a partial response, or even complete response can be seen. TIL is highly personalised and complex therapy. It requests substantial upfront investments from the hospital in expensive laboratory equipment, staff expertise and training, as well as extremely tight hospital logistics. Therefore, a randomised controlled trial including an early health economic modelling study, as part of a coverage with evidence development (CED) program, is currently ongoing.

## Health economic involvement in research: the case of The Netherlands Cancer Institute

In The Netherlands Cancer Institute (NKI), we have decided to involve health economic and technology assessment knowledge in the early stages of biomedical research in order to improve the chances of the development of innovations to be actually covered by insurance and reach the patient earlier. This started with various publications and involvement in early CED experiments on the70-gene signature, which resulted in a number of publications and on various data on the cost-effectiveness of this technology [9, 11–15]. With the start of the formal Dutch CED development program, we have decided to set up an internal ‘CED team’ and involve the technology assessment knowledge in the procedure for selection and application for these procedures and have so far been rather successful. The first example involved treatment with tumour infiltrating lymphocytes (TIL) for metastatic melanoma (Box 1). In an early phase of starting the immunotherapy programme in the NKI and of research on TIL, preliminary health economic analyses were already performed, so when the call for CED came it was relatively easy to complete the file on all the relevant aspects. In the NKI, both the coordination of the TIL trial and the execution of the health technology assessment can be performed. Combining these expertises, the necessary file elements could be completed swiftly be submitted for the CED programme selection.

In 2015, again two procedures suggested by, and based on research from the NKI, were selected by the Dutch government to be elaborated in a final proposal for CED, being high dose chemotherapy for BRCA-like triple negative breast cancer (Box 2).

Box 2. High-dose chemotherapy for BRCA1-like subgroup in triple-negative breast cancer.

In the late nineties, high-dose alkylating chemotherapy (HDAC) was provided to triple-negative breast cancer patients with little firm evidence; the high impact of the treatment on patients and fear of serious side effects lead to fierce debate within the oncology community on the appropriateness of prescribing this treatment. An RCT coordinated by the NKI showed no overall benefits [18] and after these and comparable results were published, the High Dose Chemo treatment was only provided to isolated cases.

Subgroup analysis in follow-up studies and research on genomic profiling showed that there might, however, be a group within the triple-negative population showing a BRCA1-like genetic pattern that was responding highly to HDAC. Proof of principle of first series showed promising results [19–21] and health economic modelling provided evidence for the likelihood of cost-effectiveness [7].

Intra-operative hyperthermic intraperitoneal chemotherapy (gastric HIPEC) for locally metastasised gastric carcinoma (with limited peritoneal carcinomatosis (P+) and/or tumour positive peritoneal cytology (C+)) was another example that was recently awarded to the NKI as leader of a CED project.

For both treatments (HDCT and gastric HIPEC), the same procedure was followed as earlier with the TILCED trial proposal. Leadership ensured that there was an annual central call within the NKI and a procedure for selection of promising innovations by senior clinical management within the institution. Only after careful selection a maximum of three proposals was allowed for submission. Further, it was ensured that, apart from specific cancer-related content, health economic and coverage knowledge was included in the submission of the proposals. The health economic input consisted of close involvement through literature reviews, (early stage) health economic modelling and screening on aspects of technology assessment that may be involved apart from specific economic features (such as ethical, organizational or patient related aspects; [9]). Further, it proved useful to have knowledge on national priorities and coverage criteria. This proved to be a rather successful approach as two of the three nominated procedures selected as potential CED candidates in 2015 in The Netherlands originated in the NKI.

## Conclusion and discussion

Involving health economics and health technology expertise in the early stages of research and in preparation for ‘coverage with evidence development’ (CED) experiments can be very successful in ensuring innovative technologies to reach the patient in an efficient way.

Conditions for this approach are the availability and presence of HTA and health economic expertise and knowledge on coverage procedures within (or closely available for) the institution. Oncologists have to be aware and appreciate this while drawing the project proposals for CED. Professional societies need to be involved to ensure acceptance in practice guidelines both during the CED project as well as after receiving the conclusions.

It is not absolutely necessary to have own groups or expertise on HTA, but close cooperation and scientific ‘presence’ on the site are essential. Otherwise, clinicians may not be aware of the essential and useful technicalities. When HTA experts are involved early in the translational research process, the chance of a traditional role—performing a cost-effectiveness analysis in a late stage, just before coverage is decided upon—is reduced. More innovative early stage HTA schemes are likely to evolve, which are also better fitting to the development stage of the technology. This also requires an open mind for agencies deciding on coverage, as these still tend to rely on more traditional HTA approaches.

As financial constraints are expected to become even more pressing in the future on a global level, there’s a better chance of leading their findings to the patient when they are cost effective. Early-stage involvement of ‘understanding’ HTA experts is likely to help them achieve this goal.
